# Whole-Genome Sequence of Sphingomonas sp. Strain FARSPH, a Novel Sf9 Insect Cell Culture Contaminant

**DOI:** 10.1128/MRA.01128-18

**Published:** 2018-11-01

**Authors:** Jorge Bendezu, Sandra Morales Ruiz, Ricardo Montesinos, Juana Quispe, Luis Tataje-Lavanda, Manolo Fernández-Sánchez, Manolo Fernández-Díaz

**Affiliations:** a Laboratorios de Investigación y Desarrollo, FARVET, Chincha Alta, Ica, Peru; University of California, Riverside

## Abstract

Here, we report the whole-genome sequence of Sphingomonas sp. strain FARSPH, isolated from an insect cell line as a contaminant. FARSPH shared high identity with Sphingomonas melonis and Sphingomonas aquatilis strains. Due to this finding, we recommend taking this genus into consideration for cell culture quality control.

## Abstract (precis)

Here, we report the whole-genome sequence of Sphingomonas sp. strain FARSPH, isolated from an insect cell line as a contaminant.

## ANNOUNCEMENT

A Sphingomonas sp. (Alphaproteobacteria) was identified as a mammalian cell culture contaminant which is able to produce cytopathic effects and apoptosis in these cells (1). While some members of the genus Sphingomonas showed the ability to pass through 0.2-µm filters (2), others were reported to be resistant to UV radiation and alkaline pH (3).

In this study, we present a whole-genome sequence of a Sphingomonas sp. designated strain FARSPH, which was isolated from an Sf9 insect cell line. The FARSPH strain was obtained from a white biofilm formed on the bottom of an insect cell culture flask cultivated under standard conditions (4). During the cell culture process, we observed Sf9 insect cells with large vacuoles at the perinuclear regions. These vacuoles contained motile organisms which were also visualized around the insect cells. These observed cytopathic effects were similar to those reported previously in mammalian cell culture in the presence of a Sphingomonas sp. (1).

For the isolation of bacteria, the culture medium was removed from the flaskm and the Sf9 insect cells, along with the biofilm, were washed with phosphate-buffered saline. The biofilm was scraped, collected, and centrifuged at 500 × *g* for 10 min. The pellet was resuspended in Luria broth with 0.1 mg/ml ampicillin, incubated overnight at 27°C, and harvested for DNA isolation using a phenol-chloroform protocol (5).

The whole-genome sequencing of the FARSPH strain was performed by Macrogen, Inc. (South Korea) using a PacBio RS II sequencer (Pacific Biosciences, CA), and the reads were assembled *de novo* using Canu (6). The FARSPH strain genome is composed of one circular chromosome of 3,345,474 bp (G+C content of 68%; coverage, 200×) and three circular plasmids, namely, plasmid p01 of 265,221 bp (coverage, 217×), plasmid p02 of 78,167 bp (coverage, 166×), and plasmid p03 of 72,676 bp (coverage, 175×). We identified 3,578 genes, including 3,434 coding sequences (CDSs), 60 RNA genes, 84 pseudogenes, and 1 CRISPR array using the NCBI Prokaryotic Genome Annotation Pipeline (7). From these 60 RNA genes, we identified 51 tRNAs, 6 rRNAs, and 3 noncoding RNAs (ncRNAs). Moreover, a high identity of 99% with Sphingomonas melonis DAPP-PG 224 (GenBank accession number NR_028626) and Sphingomonas aquatilis JSS-7 (GenBank accession number NR_024997) was observed. The lengths of these alignments were 1,421/1,440 bp and 1,423/1,443 bp, respectively. These identity values were observed when a BLASTn search was performed using the 16S rRNA sequence provided from the NCBI database.

Our results suggest that the FARSPH strain could be a contaminant of insect cell cultures whose genomic sequences are highly related to those of other Sphingomonas spp. isolated from sources other than cell cultures (8, 9). Therefore, this genus should be taken into consideration for quality control evaluations of cell cultures, as few contaminants (10, 11) have been reported in insect cell cultures to date.

### Data availability.

The whole-genome sequences of the FARSPH strain, plasmid p01, plasmid p02, and plasmid p03 were deposited in GenBank under the accession numbers CP029985, CP029986, CP029987, and CP029988, respectively. These sequences have been deposited in the NCBI under BioProject accession number PRJNA474545. The versions described in this paper are the first versions, CP029985.1, CP029986.1, CP029987.1, CP029988.1, and PRJNA474545, respectively.
